# On the Effect of the Nitrous Vapours in Preventing Contagion, and Arresting the Progress of Contagious Fevers

**Published:** 1804-02-01

**Authors:** Charles Gimbernat

**Affiliations:** Madrid


### *On the Effect of the* Nitrous Vapours *in preventing* Contagion, *and arresting the Progress of* contagious Fevers; *by* Mr. Charles Gimbernat, *of Madrid.*

THE public are indebted for the very useful discovery of applying nitrous vapours for the purpose of destroying contagious miasmata to the celebrated Dr. C. Smith, whose interesting work I was induced to translate into the Spanish language on the approach of a dreadful epidemic, imported into Cadiz in the year 1800 by a vessel from America, by which the whole province of Andalusia was gradually attacked, and which proved exceedingly fatal. I added to the translation, a direction for obtaining the nitrous acid pure and in white fumes, and for preventing the generation of the oxydated azotic gas or the orange-coloured fumes; this being a very essential point of the operation, as the fumes of the pure nitrious acid only, may be breathed without any trouble or danger; and I concluded ed with examining all the means hitherto proposed for purifying the atmosphere, and adding my reasons, why I thought the pure nitrous vapours to be the most preferable in inhabited places. The Spanish government, to which I had presented my work, resolved to elect two celebrated professors of Madrid, Messrs. Queralto and Serrais, who were to try the nitrous vapours in the alarming epidemic of Andalusia. These gentlemen having accepted the dangerous commission, repaired to Sevilla, where the malignant fever had carried off more than 12000 persons, and where the daily increasing mortality entirely overbalanced all medical assistance. On the day of their arrival in the city of Sevilla, Mr. Serrais was attacked by the fever, and two days after he fell a sacrifice to his zeal and the offices of humanity. Mr. Queralto, more fortunate than his colleague, luckily recovered from an attack of the epidemy, and immediately took the proper measures for employing the nitrous fumigations in one of the largest hospitals of the town, which was crowded with a great number of patients.

In order to put the use of the fumigations to a fair trial they were at first used only in one quarter of the city, and no other change was made in the mode of treatment which had hitherto been employed. The success however exceeded the most sanguine expectations, as the progress of the contagion was not only stopt from the very day the nitrous fumigations were adopted, but no patient was afterwards attacked by the fever in any part of the hospital. The fumigations proved also an excellent medicine to the patients; for all who lay in the wards, where the fumigations were continued day and night, found instant relief, and most of them recovered within a few days; instead of 12 persons dying every day in the hospital, as was usual before the application of the nitrous vapours, the number decreased soon after to only one in a day. Such a happy success could not fail of producing the greatest confidence in the fumigations, which were now used throughout the town, and soon adopted over all the province. In the space of three weeks the city of Sevilla, and in less than two months the whole of Andalusia was freed from the devastation of this dreadful epidemic. The confidence on the purifying property of the nitrous acid was pushed so far, that Mr. Cavanellas, surgeon to St. Luke's hospital, of Sevilla, having exposed the clothes of a person, who died with all the symptoms of the highest contagion, to the action of the nitrous vapours, put them upon his skin for for one day and a night, without experiencing the least contagious effect. It was generally found, that the nitrous fumes, mixed with the atmospherical air, occasioned no trouble to the breathing person, which is not the case with the simple muriatic gas, and much less with the oxygenated gas, both of which violently attack the organs of respiration; on which account they cannot be safely employed in inhabited places, but only for purifying wards from which the patients have been previously removed.

Dr. Smith's method has been altered by Mr. Queralto, this gentleman effecting the decomposition of the nitre in the cold, and not employing any heat, as has been done in the experiments made in England, and by which oxygenated azotic gas is frequently disengaged instead of nitric fumes; because, any surplus of heat is capable of separating the affinity which unites the oxygen with the azote.

This circumstance, however, is not to be apprehended, when the decomposition is undertaken in the cold, and in small quantities, whereby the nitrous acid will be volatilised, without being decomposd by means of the heat disengaged through the sulphuric acid penetrating the kali. The reason why the nitrous vapours do much less affect the organs of respiration than other acids, is perhaps because they are composed of the same constituents as the atmospherical air. It may be likewise suggested, that the nitrous vapours are of all others the best appropriated for destroying contagious miasmata, because they are not a permanent elastic fluid or a true gas, but mere fumes, volatile by means of the heat, which, when cooled, are condensed round all bodies, penetrating their interstices and surrounding their surfaces with an acid; and from this condensation the perfect neutralization of all the atoms of the contagious matter must be derived. But whatever ground these conjectures may have, it is sufficiently proved by the evidence of facts, that fumigations with nitrous acid are capable of destroying in a short time the contagious matter, by which malignant fevers are occasioned; that the organs of respiration remain unaffected; and that Spain, and perhaps Europe, derives the greatest benefit from their effect, in escaping the terrors and devastations, which so often alarm the United States of America.

